# Therapeutic effect of long-interval repeated subcutaneous administration of canine amniotic membrane-derived mesenchymal stem cells in atopic dermatitis mouse model

**DOI:** 10.1186/s12917-025-04554-w

**Published:** 2025-02-27

**Authors:** Minsoo Kim, Dasom Kong, Nam Gyo Kim, Min-Ji Kim, Hee-Yeong Kim, Jung-Ju Choi, Yu-Seung Choi, Ha-Eun Lee, Khaligh Seyedeh Farzaneh, Dohyung Kwon, Seunghee Lee, Kyung-Sun Kang

**Affiliations:** 1 The Research Institute for Veterinary Science, College of Veterinary Medicine, Seoul National University, Seoul, 08826 Republic of Korea; 2 Stem Cell and Regenerative Bioengineering Institute, Global R&D Center, Kangstem Biotech Co. Ltd., Ace Highend Tower 8, 84, Gasan digital 1-ro, Geumcheon-gu, Seoul, 08590 Republic of Korea

**Keywords:** Canine amniotic mesenchymal stem cells (cAM-MSCs), Canine, Atopic dermatitis (AD), Long-term, Calcineurin inhibitors (CNIs), Pimecrolimus (Pime), Subcutaneous administration

## Abstract

Atopic dermatitis (AD) is a chronic and inflammatory disease. According to a recent study, administration of canine MSCs is a potential therapy for immunological diseases. However, most related studies involve short-term experiments and acute atopic dermatitis animal models. Thus, studies of repeated subcutaneous injection of canine MSCs for ameliorating long-term inflammatory skin disorders have not yet been established. In this study, we evaluated the effects of long-term canine amniotic mesenchymal stem cells (cAM-MSCs) and calcineurin inhibitors (CNIs) treatments in mouse AD model for up to 8 weeks and compared the differences in therapeutic effect through canine peripheral blood mononuclear cells (PBMCs). Using a mouse model, we validated the therapeutic impact of cAM-MSCs in comparison to pimecrolimus (Pime), the most widely used CNIs, as a therapy for canine AD. Based on our results, we verified that the cAM-MSC treatment group exhibited substantially lower scores for tissue pathologic alterations, inflammatory cytokines, and dermatologic symptoms than the PBS control group. Importantly, compared with Pime, cAM-MSCs were more effective at preventing wound dysfunction and regulating mast cell activity. Additionally, we confirmed that immune modulation proteins (TGF-β1, IDO1, and COX-2) were increased in the cAM-MSCs treatment group. Furthermore, we examined the immunoregulatory effect of cAM-MSCs through the proliferation of T lymphocytes from activated canine PBMCs. As a result, cAM-MSCs suppressed the proliferative capacity of effector T cells from canine PBMCs more effectively than Pime. In conclusion, this study suggested that the cAM-MSCS could be an effective canine treatment for long-term canine AD through regeneration and immunomodulation.

**Supplementary Information:**

The online version contains supplementary material available at 10.1186/s12917-025-04554-w.

## Introduction

In canines, canine atopic dermatitis (CAD) is a chronic, inflammatory, and wound skin condition that occurs [1]. Genetic and environmental factors are important contributors to the complicated, multifaceted character of CAD [2]. Allergens from the environment, food, microbes, or insects can sensitize individuals to allergens, which can then infiltrate the skin, activate resident cells, and produce inflammatory mediators locally. Disruption of the epidermal barrier, infection with yeast or bacteria on the skin, psychogenic factors, and associated skin conditions are all contributory variables [3–5]. Surprisingly, there are notable similarities between atopic dermatitis (AD) in humans and CAD in terms of clinical and immunological characteristics, treatment modalities, and outcomes [6]. These factors make it more evident that several pathways may result in clinical symptoms of CAD. Thus, AD should be considered a term used to describe a clinical condition rather than a singular entity [7].

Several allergic disorders can induce acute sickness symptoms, including food allergies, allergic rhinitis (hay fever), atopic dermatitis (eczema), and allergic asthma. However, after being exposed to these allergens repeatedly for weeks to years, those affected also frequently experience long-term alterations in the afflicted tissues, a process known as tissue remodeling [8]. While intact allergens pass through the damaged epidermal barrier caused by AD, they can be picked, digested, and presented by antigen-presenting cells (APCs) through a process known as IgE-facilitated allergen presentation (FAP) [9]. IgE is considered to engage in IgE-FAP in addition to binding aeroallergens to trigger mast cell activation via high-affinity Fc receptors, which in turn causes histamine release to cause cutaneous inflammation [10]. On the other hand, allergens can be displayed on MHCs for T-cell identification after being phagocytosed by APCs, independent of IgE [11].

Particularly, chronic atopic dermatitis is closely related to allergies and autoimmune diseases. Furthermore, chronic atopic dermatitis causes skin damage with scratching, and immune cells of lymphatic immune circulation cause a strong immune response to the epithelial barrier [12].

In recent years, the application of stem cells has become a very promising leading-edge area of research. There is optimism about the importance of advancements in therapeutic techniques [13]. Advancements in stem cell technology have provided new opportunities for people with untreated illnesses and disorders. Recently, stem cell-based treatment has emerged as an important force in regenerative medicine [14]. In previous studies, the possibility of employing stem cell-based regenerative medicine as an alternative to traditional drug therapies has increasingly become a reality. This is largely due to the research community’s intense commitment to exploring its potential applications in a broad spectrum of diseases, including diabetes and neurodegenerative disorders [15].

Mesenchymal stem cells (MSCs) have attracted increased interest in recent decades for their potential applications in regenerative medicine. MSCs are not recognized by immune surveillance and do not causes graft rejection following transplantation because they do not express substantial histocompatibility complexes or immune-activating [16]. The capacity of MSCs to differentiate into various cell types, self-renewal, proliferate for a prolonged duration in vivo, perform paracrine effects, and exhibit immunoregulatory effects has demonstrated that they are a fascinating option for the therapy of human tissues [17]. Especially, angiogenesis, neuroprotection, and immunomodulation in tissues are impacted by the paracrine effects of MSCs [18]. Undoubtedly, several studies have demonstrated MSCs’ capacity to promote wound healing, regrowth, anti-aging, and anti-inflammatory effects in a variety of in vitro and in vivo models. Furthermore, recent advances in the cell biology of MSCs have assisted in the development of specific methods and quality assurance strategies; this eventually led to the clinical application of MSCs [19]. Unfortunately, MSC secretome-based treatment has not yet gained complete acceptance in veterinary medicine due to a lack of knowledge of the complexity of released bioactive components [20, 21]. Moreover, the long-term treatment effects of canine MSCs for CAD have not yet been established [22].

A category of immunosuppressant known as calcineurin inhibitors (CNIs) is used to treat many autoimmune diseases, such as lupus nephritis, atopic dermatitis, idiopathic inflammatory myositis, and interstitial lung disease. They also play a crucial role in immunosuppression during solid-organ transplantation [23]. T-cell activation is mediated by the cytoplasmic calcium-binding protein calcineurin. Immunophilins and calmodulin activate calcineurin itself. CNIs interact with immunophilins to regulate T-cell activation in a particular way [24, 25]. The primary mechanism of immunosuppressive action of these agents is the suppression of nuclear factor of activated T cells (NF-AT) dephosphorylation, which leads to a decrease in T lymphocyte activation and proliferation mediated by interleukin-2 (IL-2) and additional cytokine production [26]. The development of CNIs has provided veterinarians with significantly more medications for treating allergic skin illnesses [27]. In particular, 1% pimecrolimus, a category of CNIs, is typically used to treat atopic dermatitis [28].

In this study, we investigated whether cAM-MSCs can effectively alleviate atopic dermatitis symptoms while being repeatedly administered at long-term intervals to a mouse atopic dermatitis model. Especially, we compared clinical availability, safety and differences between cAM-MSCs and commercially available drugs.

## Materials and methods

### Animal test

Six-week-old male BALB/c mice were purchased from DBL CO. Ltd. (Eumseong-gun, Chungcheongbuk-do, Republic of Korea). All animal study was authorized by Seoul National University’s Institute of Laboratory Animal Resources (Approval No. SNU-210930-6-2). Mice were stabilized and assigned randomly for seven days (4 mouse each group, total 16 mice of 4 groups). To develop AD-like skin lesions, upper back hair of Male BALB/c mice was trimmed with an electric trimmer and repeatedly administered 2% 1-Chloro-2, 4-dinitrochlorobenzene (DNCB; Sigma-Aldrich, Germany, Catalog number: 237329) mixed with acetone (JUNSEI, Tokyo, Japan, Catalog number: 11265S0380)/olive oil(Sigma-Aldrich, Germany, Catalog number: O1514) (3:1). After that, three times a week for seven weeks, 0.2% of DNCB was applied to the dry skin. Mice’s backs were periodically shaved again to get rid of any fur growth. Subcutaneous injections of 1.5 × 10^6^ canine AM-MSCs of passage 6 with phosphate-buffered saline (PBS) or PBS alone were given to mice on days 14, 28, and 42. Then, we applied 1% pimecrolimus (Elidel cream; Novartis, USA, Catalog number: 21–302) to the AD skin of the mice with a cotton swap daily. On day 56, mice were sacrificed using CO_2_ euthanasia chamber (JEUNG DO BIO & PLANT CO., LTD, Seoul, Republic of Korea, Catalog number: JD-C-107 M) for 2 ~ 3 min, and the dorsal skin and spleen were taken. The severity assessments of erythema, scarring/dryness, edema, and erosion (none, 0; mild, 1; moderate, 2; and severe, 3) were added up to determine the dermatological scoring index. Blinded scoring was conducted on days 14, 28, 42, and 56.

### Cell culture

We isolated canine AM-MSCs as previously described [29]. Every experiment detailed here was authorized by the Institute of Laboratory Animal Resources (Seoul National University, SNU-210930-6-2, and Republic of Korea) and complied with its rules. The samples of amniotic membranes (*n* = 15) came from various specimens, and thus, this study did not include sample pooling. After parturition, placentas were collected and were provided by Smile Veterinary Clinic (Yongin-si, Gyeonggi-do, and Republic of Korea) in 50 ml conical tube with PBS comprising primocin 1% (InvivoGen, San Diego, USA, Catalog number: ant-pm-05). To isolate MSCs, we separated the amniotic membrane from the placenta. 2 mg/ml collagenase type II (Gibco, Saint Louis, USA, Catalog number: 17101015) was used to digest the minced tissues for around 3–4 h. at 37 °C in 5% CO_2_ incubator (PHCbi, Tokyo, Japan, Catalog number: MCO-170AIC). To eliminate any debris, we passed through the tissue with a 40µM cell strainer (SPL, Pocheon-si, and Republic of Korea, catalog number: 93040). Then, we performed those centrifugations on the samples at 1000 rpm for five minutes three times. After that, pellet was suspended in KSB-3 (Kang Stem Biotech, Gwangmyeong-si, Republic of Korea, Catalog number: K3901) with 10% fetal bovine serum (FBS; Gibco, Saint Louis, USA, Catalog number: 26140079). We replaced the culture medium daily and grew cells in 5% CO_2_ incubator with a humidified environment (we used cAM-MSCs cell line #1).

### H&E (Hematoxylin and eosin) staining

Hematoxylin (Merck, Darmstadt, Germany, Catalog number: 1.09253.0500) was used staining the slides for six minutes. After that, running water was used to wash them. The slides were then dipped three times in distilled water after being stained with eosin (MUTO, Tokyo, Japan, Catalog number: 32002) for one minute. The stained slides were three times immersed in varying concentrations of 70, 80, 90, and 100% ethanol (Duksan, Republic of korea, Catalog number: 64-17-5) for dehydration. An hour was spent incubating the stained slides in xylene. After that, the slides were covered using Canada balsam (Junsei, Tokyo, Japan, Catalog number: 8007-47-4). The Canada balsam-sealed stained slides were left to dry overnight.

### Toluidine blue staining

To make the toluidine blue stock solution, 100 ml of 70% ethanol was combined with 1 g of Toluidine Blue O (Sigma-Aldrich, Darmstadt, Germany, Catalog number: T3260). After mixing, the toluidine blue stock solution was dissolved. To stain mast cells, thoroughly combine 45 ml of 1% sodium chloride (pH 2.3 to 2.5, SAMCHUN, seoul, republic of Korea, Catalog number: S0484) with 5 ml of Toluidine Blue stock solution. Three xylene washes for ten minutes were performed on paraffin tissues on slides coated with saline. Sections of the slides were hydrated for ten minutes with distilled water. After staining sections for two to three minutes in toluidine blue working solution, rinse them three times with distilled water. To dehydrate the slides, they were dipped three times each in 70, 80, 90, and 100% ethanol. An hour was spent incubating the stained slides in xylene. After that, the slides were covered using Canada balsam. The Canada balsam-sealed stained slides were left to dry overnight.

### Real-time PCR

TRIzol reagent (Invitrogen, Carlsbad, USA, Catalog number: 15596018) was used to extract total RNA from the tissue after the prescribed protocols. cDNA was generated from extracted RNA and detected by real-time qPCR. Quant Studio Design and Analysis Software v1.4 was used to examine the data. All genes’ expression level comparisons were done by normalizing each level by GAPDH. For every gene, at least three separate studies have been conducted. The primers utilized in real-time qPCR are listed in Supplementary Table 1.

### Immunohistochemistry (IHC)

Slides were incubated with PBS containing 0.25% Triton-X 100 (PBST; Sigma-Aldrich, Darmstadt, Germany, Catalog number: X100) for 30 min. The slides were blocked using a blocking solution containing 5% normal goat serum in 0.025% PBST. After diluting primary antibodies in blocking solution, the slides were covered and stored at 4 °C overnight. After diluting secondary antibodies in 0.025% PBST, the sections were exposed to them for one hour at room temperature. Secondary antibodies conjugated to Alexa Fluor 488 (Invitrogen, Carlsbad, USA, Catalog number: A20181) and 594 (Invitrogen, Carlsbad, USA, Catalog number: A20185) were used at a dilution of 1:500. After being diluted 1:1000 in 0.025% PBST, DAPI (Invitrogen, Catalog number: D3571) was applied to the slides and left for ten minutes. Finally, the stained slides were then covered with fluorescent mounting solution and allowed to air dry. A confocal microscope took three pictures of each slide. Supplementary Table 2 provides an inventory of antibodies used in immunostaining methods.

### Mixed lymphocyte reaction (MLR) assay

We carefully filled the upper layer of a 50 ml conical tube (Kirgen, Shanghai, China, Catalog number: KG2811) with 15 ml of Ficoll (cytiva, USA, Catalog number: 17144002) and 15 ml of canine blood/PBS (1:1). Samples were then centrifuged for 30 min at 400Xg. We gently transferred the substance to a clean bench and gathered the PBMC layer. To perform activating Tcell from PBMC, we treated concanavalin A 5µg/ml (conA; InvivoGen, San Diego, USA, Catalog number: inh-cona-2) to 1 × 10^6^ PBMCs (bottom) with RPMI 1640 (Gibco, Saint Louis, USA, Catalog number: 11875093). Then, co-culturing 1 × 10^5^cAM-MSCs (upper) or treating pimecrorlimus 100ng/ml (Sigma-Aldrich, Darmstadt, Germany, Catalog number: SML1437) through a 0.4 μm pore size Trans well (CORNING, Corning, USA, Catalog number: 3460) on 24 multi-well dishes on four and eight days. After that, utilizing Tecan (Life science, USA), samples were gathered for the Cell Proliferation ELISA and BrdU (colorimetric) tests (Roche, Mannheim, Germany, Catalog number: 11647229001).

### Fluorescence-activated cell sorting (FACS)

Applying a FACS analyzer equipped with a flowzo_V10 (BD Science, USA), PBMCs were subjected to flow cytometry analysis. First, the cells were washed three times with PBS. The cells were stained with CCR7 (C-C chemokine receptor type 7; clone: 3D12, 1:100 dilution, BD science, USA, Catalog number: 552176), cluster of differentiation 45ra (CD45ra; clone: HI100, 1:500 dilution, BD science, Catalog number: 550855), and CD62L (L-selectin; clone: FMC46, 1:100 dilution, Novus bio, Catalog number: NB100-65388) for two hours at 4 °C under blocking light. Paraformaldehyde (PFA; Sigma-Aldrich, Germany, Catalog number: 158127) was used to fix the cells for 10 min. After being the cells suspended in 300–500 µl PBS, the cells were examined using flowzo_V10.

### Statistical analysis

All values are presented as the mean ± standard deviation (SD). ANOVA or the 2-tailed Student’s t-test were used for statistical analysis, and GraphPad Prism version 5.0 (GraphPad Software, San Diego, USA) was used for multigroup comparisons. The Newman-Keuls post hoc test was then used. The legends for the figures define statistical significance.

## Results

### Long-interval repeated subcutaneous administration of cAM-MSCs ameliorated the symptoms of the DNCB-induced atopic dermatitis mouse model on day 56

**Fig. 1:**
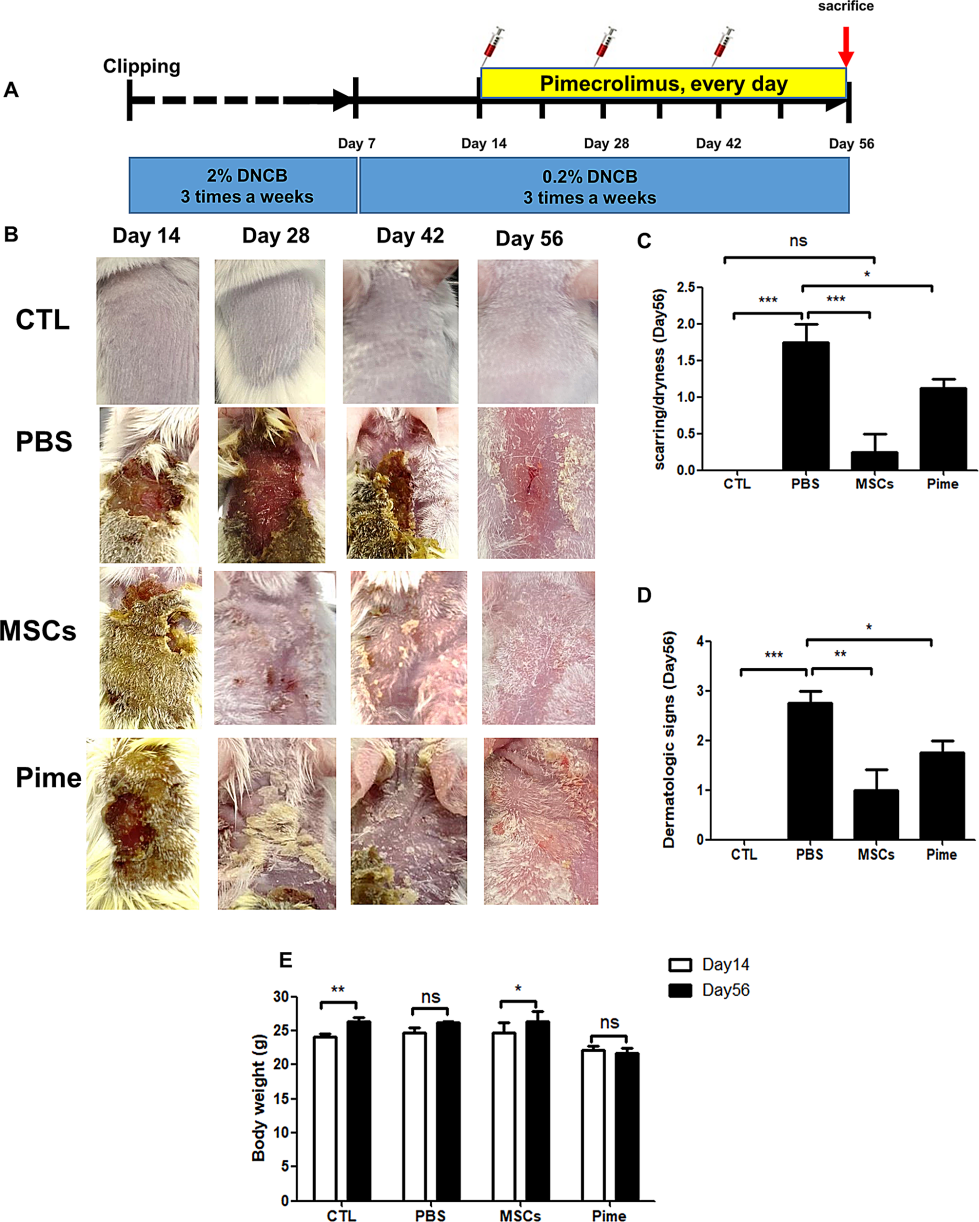
Treatments with cAM-MSCs effectively reduced the symptoms of the experimental DNCB-induced atopic dermatitis mouse model on Days 28 and 56. (**A**) Scheme of the experimental atopic dermatitis mouse model, including the time point at which DNCB was administered. (**B**) Representative images of AD mice on Days 14, 28, 42 and 56 after treatment with cAM-MSCs (MSCs); negative control (CTL); pimecrolimus (Pime); and PBS control (PBS). (**C**) Scoring of scarring and dryness signs for CTL, PBS, MSCs and Pime on Day 56 (none, 0; mild, 1; moderate, 2; and severe, 3). *n* = 4 in each group; mean ± SD; * *p* < 0.05, *** *p* < 0.001, n.s. not significantly different versus the control. (**D**) Scoring of dermatologic signs for DNCB-induced mice in each group on Day 56 *n* = 4; mean ± SD, * *p* < 0.05, ** *p* < 0.01, *** *p* < 0.001. (**E**) Body weights of atopic dermatitis mouse model in each group on Day 14 and Day 56; *n* = 4; mean ± SD, * *p* < 0.05, ** *p* < 0.01 n.s. not significantly different versus the control

MSCs are known to be effective as a single-dose treatment without any major side effects in acute AD, so previous studies have presented a schematic of the short-term AD model [30, 31]. In this study, we confirmed the long-term efficacy of cAM-MSCs and their differences from commercial drugs. Thus, one group of DNCB-induced AD mice was administered subcutaneous injections of 1.5 × 10^6^ canine AM-MSCs with PBS were given to mice AD dermal skin on days 14, 28, and 42, and another group was administered 1% pimecrolimus ointment daily. In this reason, we designed for the long-term experimental scheme shown in Fig. 1A. In this experiment, the cAM-MSCs treatment group showed significant improvements in the severity of symptoms (scarring/dryness score: 0.3 ± 0.5, erythema score: 0.8 ± 0.5, edema score: 0, erosion scores: 0; Fig. 1B, C, supplement Fig. 1. D, E and F) and the clinical score (1.0 ± 0.8; Fig. 1D) than PBS control group (scarring/dryness; score: 1.8 ± 0.5, erythema score: 1, edema score: 0, erosion scores: 0, clinical score: 2.8 ± 0.5) on day 56 (Fig. 1B, C, D, supplement Fig. 1D, E and F). Next, our data showed that significant weight gain was observed in the control (CTL) 24.1 ± 0.8 g on day 14 to 26.4 ± 1.2 g on day 56 and MSC treatment group 24.8 ± 2.8 g on day 14 to 26.4 ± 2.9 g on day56, but no significant weight gain was observed in the PBS control group and the pimecrolimus treatment group (Fig. 1E). Moreover, spleen length and weight were not increased in the cAM-MSCs treatment group (Supplemental Fig. 1A-C). These results showed that, on Day 56 of an AD mouse model, cAM-MSCs were more effective than PBS control at reducing visual symptoms and gross scores without causing any significant side effects.

### The dermal skin of the mouse AD model was evaluated through histological analysis on day 56

**Fig. 2:**
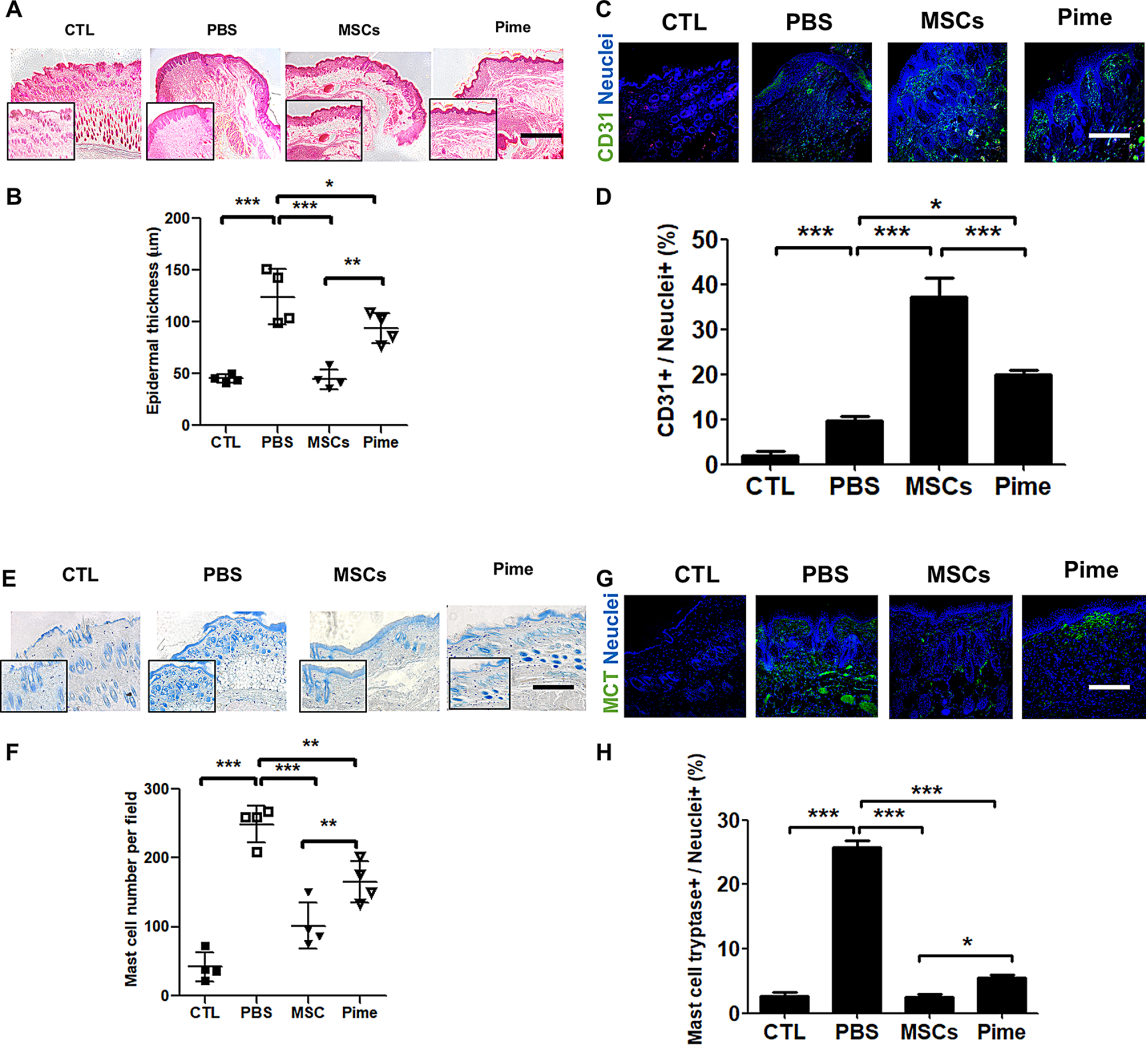
Mouse skin was evaluated though Histological analysis. (**A**,** B**) (**A**) Representative images of Hematoxylin and eosin (H&E) staining in CTL, PBS, MSCs and Pime. Scale bars, 500 μm. (**B**) Quantification of epidermal thickness. *n* = 4 for each slide, mean ± SD; * *p* < 0.05, ** *p* < 0.01, *** *p* < 0.001. (**C**,** D**) (**C**) Representative confocal images of CD31-positive cells (green) and nuclei (blue) stained with endothelial markers in the CTL, PBS, MSCs and Pime groups. Scale bars, 200 μm. (**D**) Quantification of marker CD31-positive cells. *n* = 3 for each slide, mean ± SD; * *p* < 0.05, *** *p* < 0.001. (**E**,** F**) (**E**) Representative images of Toluidine blue staining in CTL, PBS, MSCs and Pime. Scale bars, 500 μm. (**F**) Quantification of mast cell numbers per field. mean ± SD; ** *p* < 0.01, *** *p* < 0.001. (**G**,** H**) (**G**) Representative confocal images of MCT-positive cells (green) and nuclei (blue) stained with mast cell activation markers in CTL, PBS, MSCs and Pime. Scale bars, 200 μm. (**H**) Quantification of marker MCT-positive cells. *n* = 3 for each slide, mean ± SD; * *p* < 0.05, *** *p* < 0.001

To investigate the thinness of the dermal skin, we conducted a histological assessment. The cAM-MSCs treatment (45.2 ± 3.9 μm) and pimecrolimus treatment group (94.0 ± 14.7 μm) had significantly reduced epidermal thickness in the dorsal skin compared to PBS control group (124.2 ± 26.6 μm). Especially, epidermal thicknesses in the cAM-MSCs treatment group were lower than the pimecrolimus treatment group (Fig. 2A, B).

Many studies have shown that the paracrine effect of growth factors by MSC transplantation can significantly enhance cutaneous wound healing via angiogenesis [32, 33]. Especially, endothelial cells display the cell adhesion molecule CD31, often referred to as Platelet/endothelial cell adhesion molecule-1 (PECAM-1), widely on their surface. To investigate endothelial marker-related regeneration of vessels in dermal skin of AD mice, we stained cross-sections of mouse dermal skin for CD31. (Fig. 2C, D). We found that the number of CD31-positive cells was increased in the 37.2 ± 7.3% cAM-MSCs and 20.0 ± 1.7% pimecrolimus treatment group than in the 9.7%±1.6% PBS control group. In particular, CD31-positive cells in cAM-MSC treatment group had significantly more increased than the pimecrolimus treatment group.

Next, we evaluated mast cell infiltration in mouse dermal skin with AD. Consequently, compared to the PBS control group (248.8 ± 26.8 per field) and pimecrolimus treatment group (165.5 ± 29.9 per field), mast cells were considerably reduced in the cAM-MSCs treatment group (101.5 ± 33.4 per field) (Fig. 2E, F). Moreover, we stained and quantified marker-positive cells for the mast cell activation marker mast cell tryptase (MCT) in the dermal layer of the mouse skin (Fig. 2G, H). In this result, the cAM-MSCs treatment group (2.5 ± 0.7%) had significantly decreased MCT-positive cells than PBS control (25.8 ± 1.8%) and pimecrolimus (5.5 ± 0.7%). Our data confirmed that cAM-MSCs effectively improve wound dysfunction and regulate mast cell activity more PBS control and pimecrolimus treatment group.

### The anti-inflammatory activities for type 2 inflammation of cAM-MSCs reduced AD on mouse skin

**Fig. 3:**
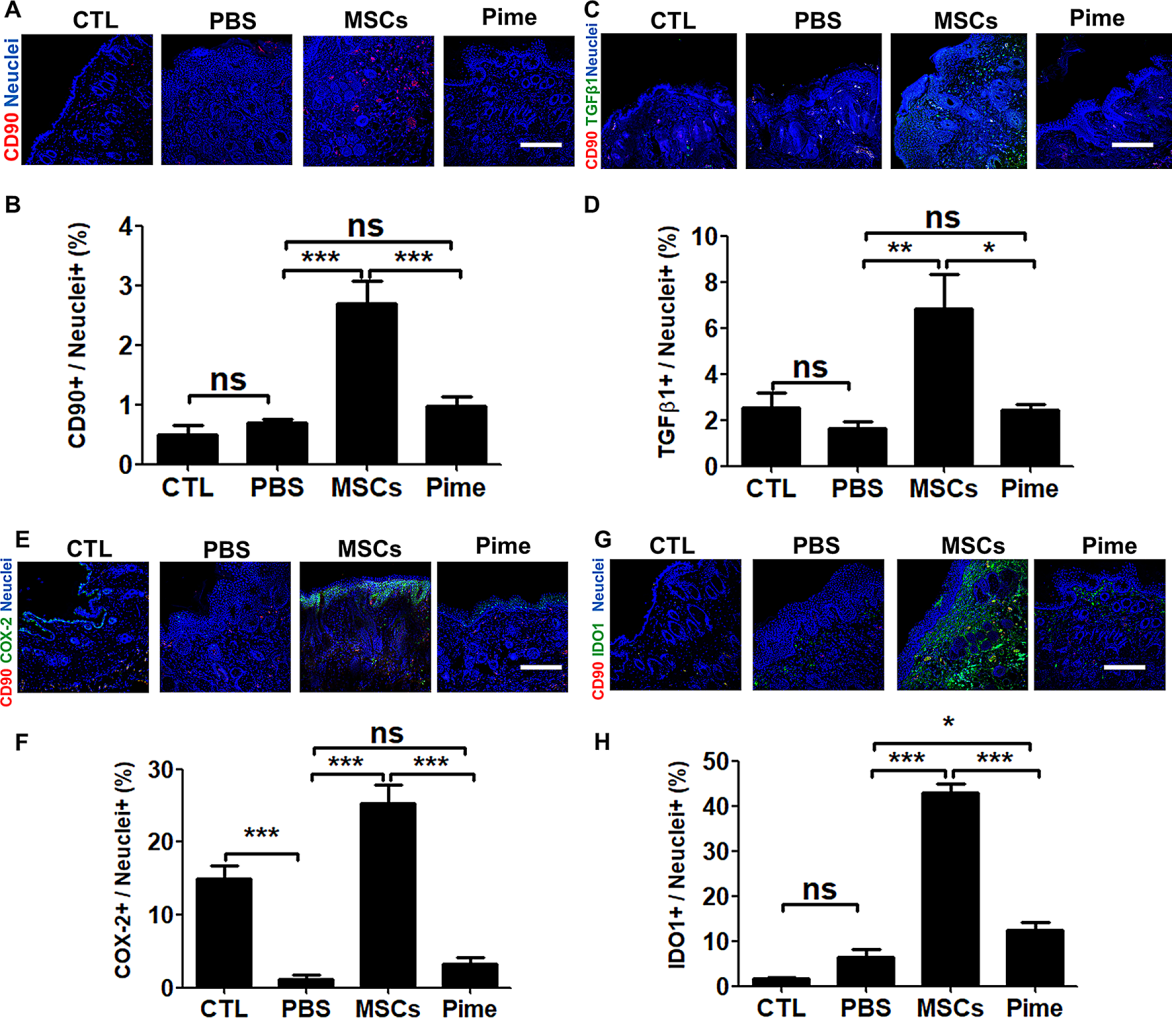
Immunomodulation proteins of cAM-MSCs in mice skin as determined by immunostaining. (**A**,** B**) (**A**) Representative confocal image showing canine MSC-positive CD90 cells (red) and nuclei (blue). Scale bars, 200 μm. (**B**) Quantification of marker CD90-positive cells. *n* = 3 for each slide, mean ± SD; *** *p* < 0.001, n.s. not significantly different from control. (**C**,** D**) (**C**) Confocal image showing the immunomodulation marker TGFβ1 (green), CD90-positive canine MSCs (red) and nuclei (blue); scale bars, 200 μm. (**D**) Quantification of the marker TGFβ1-positive cells. *n* = 3 for each slide, mean ± SD; * *p* < 0.05, ** *p* < 0.01, n.s. not significantly different from the control. (**E**,** F**) (**E**) Confocal image showing the immunomodulation marker COX-2 (green), CD90-positive canine MSCs (red) and nuclei (blue); scale bars, 200 μm. (**F**) Quantification of COX-2-positive cells. *n* = 3 for each slide, mean ± SD; *** *p* < 0.001, n.s., not significantly different versus the control. (**G**,** H**) (**G**) Confocal image showing the immunomodulation marker IDO1 (green) and CD90-positive canine MSCs (red) and nuclei (blue); scale bars, 200 μm. (**H**) Quantification of marker IDO1-positive cells. *n* = 3 for each slide, mean ± SD; * *p* < 0.05, *** *p* < 0.001, n.s. not significantly different from the control

Next, we stained and quantified for canine MSCs positive surface marker CD90 in mouse dermal skin (Fig. 3A, B). These results showed that canine CD90-positive cells were observed in cAM-MSCs treatment group (2.7 ± 0.6%). On the contrary, other groups detected low CD90-positive cells (CTL: 0.5 ± 0.35%, PBS control: 0.7 ± 0.07 and Pime: 0.97 ± 0.35%). In this data, we confirmed that CD90-positive cells were significantly in mice dermal skin of cAM-MSCs treatment group than other groups. To investigate the anti-inflammatory effect by cAM-MSCs, we examined the transcriptional changes linked to inflammatory genes to assess inflammation in the dermal skin of mice with AD Using real-time PCR (Supplemental Fig. 2A, B). The immune inflammatory factors IL-4, a major cytokine from Th2 cells, and IL-6, a major pro-inflammatory cytokine, were significantly decreased after treatment with cAM-MSCs than PBS control.

To investigate the secretion of immunomodulatory proteins in mouse dermal skin, we stained TGF-β1, COX-2 and IDO1 (Fig. 3C-H). The results of this experiment showed that the cAM-MSCs treatment group significant increase in 6.8 ± 2.6% TGF-β1, 25.3 ± 4.4% COX-2, and 43.0 ± 3.2% IDO1 than PBS control and pimecrolimus group. These data showed that cAM-MSCs reduced inflammatory factors in mouse dermal skin by immunomodulation proteins.

### Effective T cell activation from canine PBMCs is reduced by cAM-MSCs

**Fig. 4:**
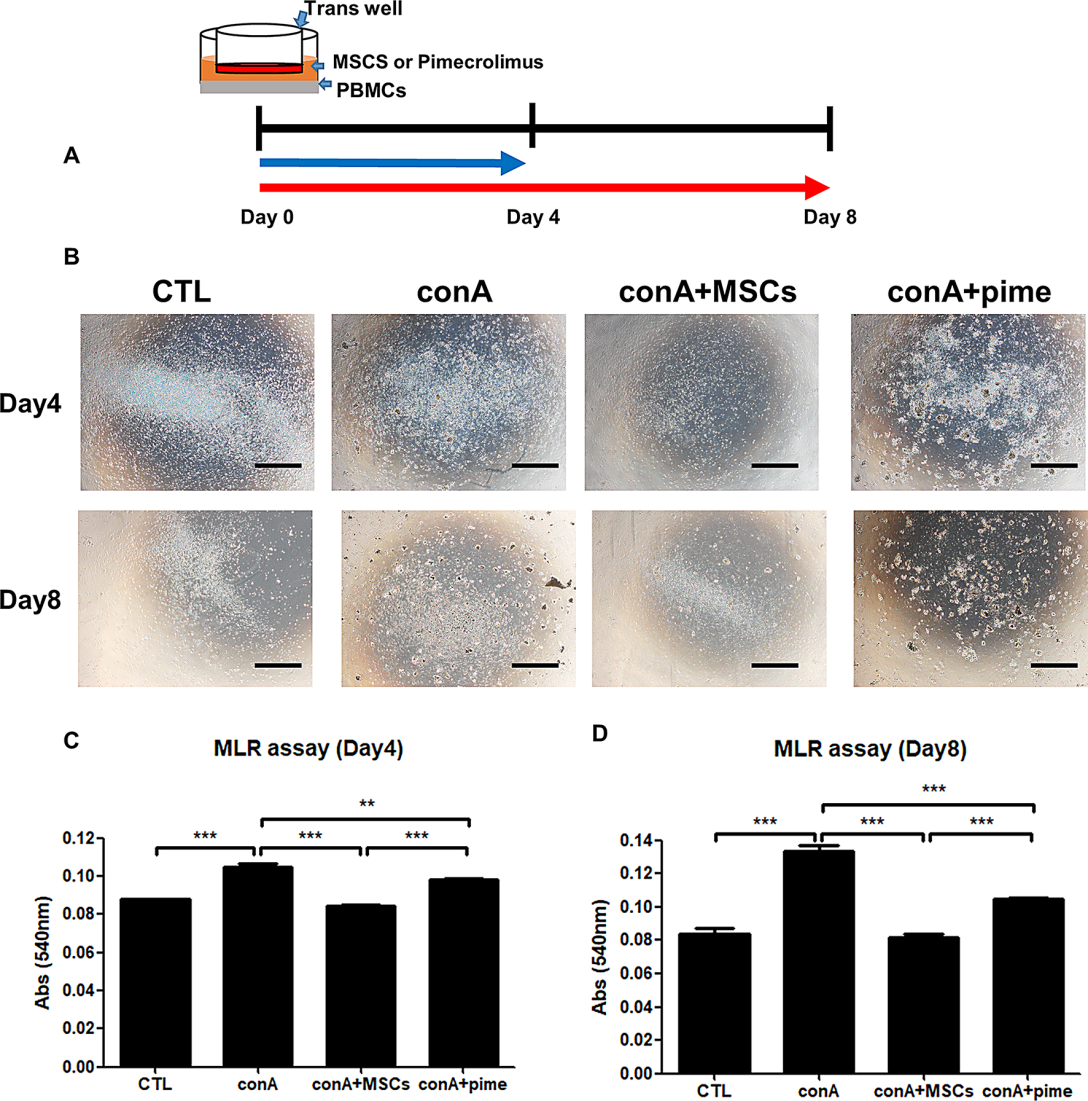
Immunomodulation of cAM-MSCs on Days 4 and 8 in vitro. (**A**) Schematic overview of the use co-culture for immunemodulation. (**B**) Representative phase-contrast image of CTL (PBMCs), PBMCs were treated with concanavalin A (conA; 5 μm/ml), conA combined with MSCs (1 × 10^5^cells), conA combined with pimecrolimus (Pime; 100 ng/ml) on Days 4 (upper) and 8 (bottom). Scale bars, 200 μm. (**C**) On Day 4 of the MLR assay, lymphocytes were quantified in triplicate using absorbance at 540 nm. mean ± SD; ** *p* < 0.01, *** *p* < 0.001. (**D**) On Day 8 of the MLR assay, lymphocytes were quantified in triplicate using absorbance at 540 nm, mean ± SD; *** *p* < 0.001

To investigate comparing the short-term (Day 4) and long-term (Day 8) immunomodulatory effects of cAM-MSCs in canine PBMCs, we designed scheme of a mixed lymphocyte reaction (MLR) assay (Fig. 4A). To perform activating Tcell from PBMC, we treated concanavalin A 5µg/ml (conA) to 1 × 10^6^ canine PBMCs (bottom) with RPMI 1640. Then, co-culturing 1 × 10^5^cAM-MSCs (upper) or treating pimecrolimus (Pime) 100ng/ml through a trans well on four and eight days. After the experiment, we observed the morphology of the cells (Fig. 4B). This result showed a similar immune response and cell differentiation in each group (CTL, conA, conA + MSCs and conA + Pime) on day 4 and day 8. Furthermore, we measured the efficiency of immune modulation in canine PBMCs (Fig. 4C, D). As a result, we confirmed that, both in the short and long terms, cAM-MSCs inhibited the activation of lymphocytes from canine PBMCs.

### cAM-MSCs inhibited effector T-cell activation in canine PBMCs

**Fig. 5:**
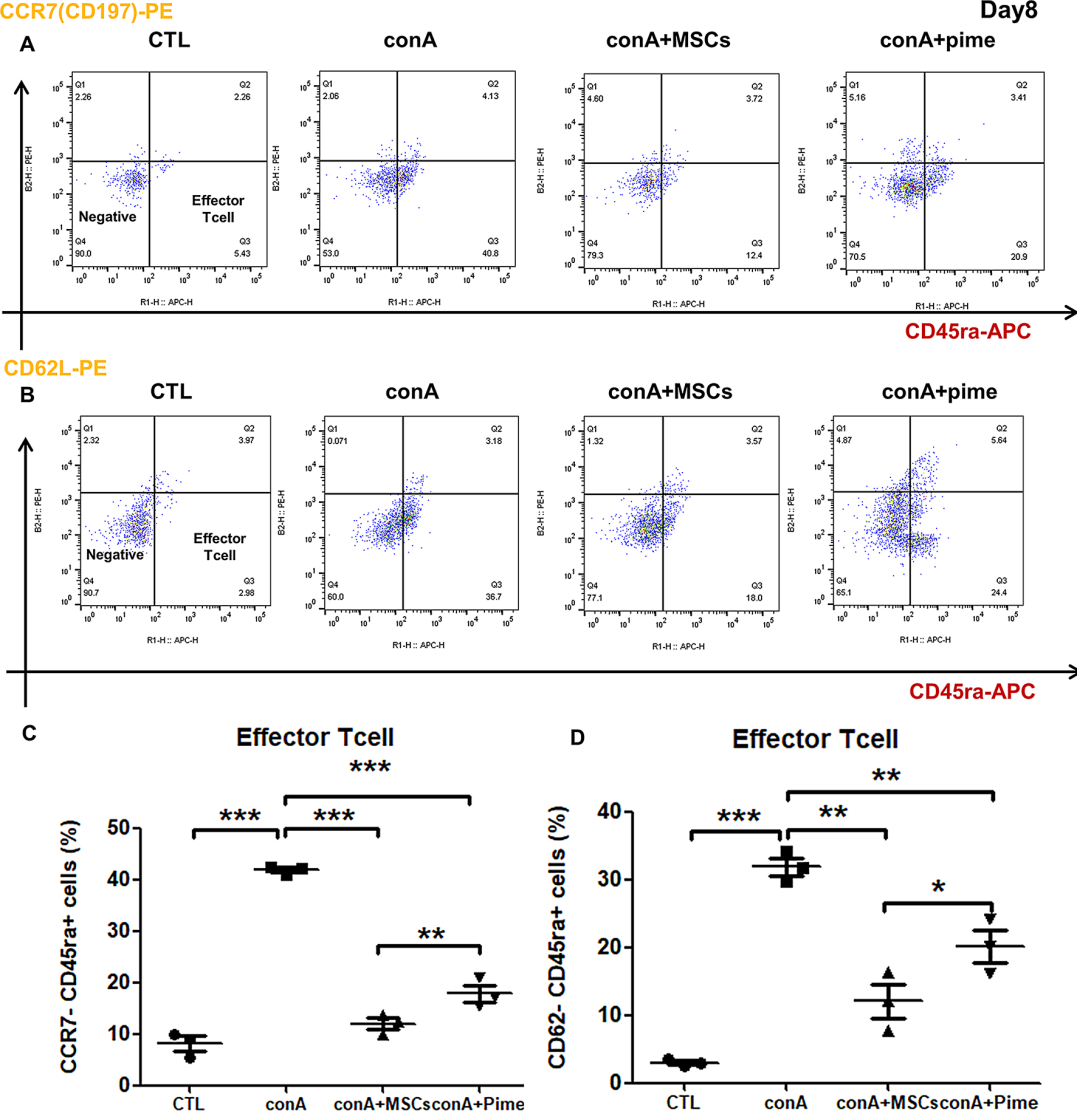
Expression profiles of CD markers in T cells determined by flow cytometry analysis after stimulation of canine PBMCs with conA. (**A**) Representative FACs image of CTL, conA, conA + MSCs and ConA + Pime. PBMCs were treated with concanavalin A (conA; 5 μm/ml), conA combined with MSCs (1 × 10^5^cells), conA combined with pimecrolimus (Pime; 100 ng/ml) until Day 8. The markers used were CCR7 (CD197) and CD45ra. Three independent analyses were conducted. (**B**) Representative FACs image of CTL, conA, conA + MSCs and ConA + Pime. PBMCs were treated with concanavalin A (conA; 5 μm/ml), conA combined with MSCs (1 × 10^5^cells), conA combined with pimecrolimus (Pime; 100 ng/ml) until Day 8. The markers used were CD62L and CD45ra. Three independent analyses were conducted. (**C**) Quantification of CCR7-negative and CD45ra-positive cells through flow cytometry analysis in triplicate. mean ± SD; ** *p* < 0.01, *** *p* < 0.001. (**D**) Quantification of CD62L-negative and CD45ra-positive cells through flow cytometry analysis in triplicate. mean ± SD; * *p* < 0.05, ** *p* < 0.01, *** *p* < 0.001

To further study for the long-term (Day 8) immunomodulatory effects, we performed FACS analysis. These results showed that the numbers of CCR7-/CD45ra + cells, surface molecules of effector-Tcell, were reduced after co-culturing canine PBMCs with cAM-MSCs (12.4%) than conA only (40.8%) (Fig. 5A, C). Additionally, we observed that CD62L-/CD45ra + cells were also significant decreased with cAM-MSCs (18.0%) than conA only (36.7%) (Fig. 5B, D). According to these results, cAM-MSCs reduced effector-Tcell from canine PBMCs. Moreover, effector T cell activation was inhibited by cAM-MSCs more effectively than pimecrolimus (20.9% CCR7-/CD45ra + cells, 24.4% CD62L-/CD45ra + cells).

## Discussion

Similar to human diseases, canine atopic dermatitis is defined as a chronic, inflammatory skin disease that has high morbidity. Given that this incurable disease requires regular therapy and management, it not only decreases the quality of life on both companion animals and their owners but also increase economic burden which can result the increase of anima abandonment [7, 34]. Recent investigations have concentrated on the therapeutic applications of MSCs immunomodulation, with an increasing number of studies demonstrating the potential of MSC-based therapies in the management of atopic dermatitis in canines [35–37]. In our study, we compared to repeated administration of pimecrolimus, a commercialized chemical drug, with long-interval dosing of MSCs. Then, we investigated the efficacy, side effects, and mechanisms of action each treatment.

This study confirmed the possibility of long-term therapeutic efficacy by administering canine amniotic membrane stem cells to a mouse AD model. However, cross-species animal testing using animal models has limitations in clearly identifying cell therapy products. Therefore, it is necessary to conduct additional research on the clear long-term efficacy of stem cells in canine atopic dermatitis patients.

We performed long-interval repeated administration and the experiment was conducted twice the typical AD mouse animal experiment period of 28 days. Recently, several papers have shown that MSCs can generate long-term efficiency of therapy even after they are lost in the region [38–40]. Therefore, after cell administration, it is supposed that the efficiency of therapy from MSCs maintained the environment of the immune response and increased the adaptation of immune cells. However, further studies are required after cell therapy in the AD model.

In this study, BALB/c mice were used as experimental models for DNCB-induced atopic dermatitis. Typically, NC/Nga mouse is the most widely used model of atopic dermatitis in humans [41, 42]. On the other hand, Based on some research, it is more suitable to apply repeated DNCB to BALB/c mice rather than the NC/Nga model in exhibiting IL-4 related immune response [43]. In this study, we confirmed that conditions inducing atopic dermatitis can clearly trigger symptoms, including histological changes in lesions and inflammatory cell infiltration in BALB/c mice. These pathological changes were observed to persist until day 56 significantly compared to those in the normal control group.

Pimecrolimus are representative of topical calcineurin inhibitors that are both safe and effective in treating atopic dermatitis [44]. However, in companion animals, the most often reported adverse effect in human researches is skin irritation, and gastrointestinal issues might result from licking or ingesting topical ointment [45]. Moreover, in 2005, due to the absence of long-term safety evidence and the possibility of skin cancer development, the US FDA’s Pediatric Advisory Committee issued a “black box” warning for tacrolimus ointment and pimecrolimus cream. Unfortunately, this issue is an ongoing controversy [46, 47]. For this reason, the FDA has warned that this medicine should not be used long-term [48]. In this study, we confirmed that three administrations of cAM-MSCs maintained therapeutic effectiveness for 42 days more than an effect of pimecrolimus daily without stress or suffering for companion animals. Additionally, we observed no significant negative impact on body weight. These results suggest that cAM-MSCs could serve as an effective stem cell therapy, potentially overcoming the limitations associated with chemical drugs.

When chronic autoimmune and allergic reactions occur in the skin epithelial tissue, not only continuous itching but also wounds are induced due to continuous scratching [1, 7, 12]. To attenuate the immune response, it is essential to address the localized inflammatory reactions. Notably, to reduce stress and pain in animals, long-term cellular treatments are more beneficial compared to chemical topical treatments, which offer short-term effects. Local administration of MSCs, which are effective as therapeutic agents, can produce effective anti-inflammatory effects [38, 49].

Based on the general characteristics of MSCs, MSCs carry out their functions by secreting bioactive substances that encourage an environment and culture conducive to the repair and regeneration of injured tissues [18]. In this study, we showed that cAM-MSCs can exhibit therapeutic efficacy by inducing skin regeneration (Figs. 1B and C and 2A to D). However, further research is necessary to evaluate epithelial barrier integrity in vivo and to assess the regenerative efficacy of keratinocytes through in vitro tests.

Several therapeutic uses of MSCs have been made possible by their remarkable biological characteristics and potent immunoregulatory effects [49]. The immune modulation factors that are implicated in the secretome include TGFβ-1, IDO1, COX-2 and PGE2 [50]. In our study, we investigated whether the applied MSCs upregulated the known immunomodulatory proteins (Fig. 3C to H). In previous research [29], by a single subcutaneous injection, the paracrine effect of MSCs was shown to inhibit mast cells and promote repair of the disrobed skin barrier. In this study, we confirmed that repeated administration of MSCs produces a consistent effect. These effects were associated with the secretion and production of TGFβ-1, IDO1, and COX-2 by the MSCs. on the other hand, we confirmed the association of PGE2 in immune regulation through COX-2, which directly correlates with the product of PGE2 [51]. Consequently, we confirmed to expression level of COX-2 in Fig. 3E and F.

The two most significant specialized antigen-presentation cells (APCs) in the skin are langerhans cells and dendritic cells (DCs). These cells function as gatekeepers, delivering captured antigens on major histocompatibility complex (MHC) proteins to naïve T lymphocytes. The latter occurs in lymph nodes that drain skin and is mediated by certain chemokine receptors (CCR4, CCR10, and CCR8). Thus, T cells are primed for skin-homing and activated [52, 53]. Subsequently, T cells can initiate diverse differentiation processes based on their environment. Specific antigens can cause T cells to proliferate clonally, produce cytokines, and activate neighboring cells (such as B cells) or other immune cells (such as keratinocytes) in their immediate surroundings. Proinflammatory chemokines and cytokines are produced locally during inflammation and promote T-cell recruitment into the skin [54]. Our result showed that cAM-MSCs effectively reduced IL-6, a major cytokines of proinflammatory, and reduced spleen size and weight compared to PBS control group.

In particular, skin-tropic effector T-cell clones are produced in lymph nodes that drain the skin from naïve T-cell progenitors that have been activated by antigen-presenting DCs [55, 56]. Under these circumstances, clonally expanded effector T cells are generated; these cells migrate to the skin and concentrate most strongly at the site of initial antigen contact [57]. In this study, we proved that cAM-MSCs regulate the immune system’s reaction to long-interval AD treatment. Our results indicate that effector T cell proliferation is inhibited by the immunosuppressive property of cAM-MSCs. According to the previous studies, the regulation of effector T cells is associated with the regulation of dendritic cell activation as well as with the direct suppression of effector T cell activation by MSCs [58]. However, further study is required to confirm a more accurate mechanism on the regulation of effector T cells by cAM-MSCs.

Type 2 helper T (Th2) cell-mediated immune responses play a critical role in the development of AD, and epidermal dysfunction has a detrimental impact on the pathophysiology of this disease. These findings are supported by recent successful therapies for AD [57]. Undoubtedly, type 2 Th2-mediated immune responses and barrier dysfunction are the targets of novel medicines that might increase the number of therapeutic choices available to individuals with AD [59]. Based on our results, cAM-MSCs effectively reduced IL-4, a major cytokine from Th2 cells, and suppressed the activation of mast cells during early allergic reactions. Especially, histological changes involving the regeneration of vascular endothelial cells were observed in the cAM-MSCs administration group, and that was not verified in other chemical drugs. Taken together, cAM-MSCs effectively alleviate symptoms of atopic dermatitis by reducing the activity of mast cells and effector T cells including Th2 cells, and demonstrating distinct regenerative effects. Furthermore, it was confirmed that they maintain a high level of symptom improvements over the long-term without any side effects.

In conclusion, we applied repeated subcutaneous administration of cAM-MSCs to long-term canine atopic dermatitis. First, we evaluated the effects of long-term cAM-MSCs and pimecrolimus treatments in mouse AD model for up to 8 weeks. Based on this result, cAM-MSCs significantly reduced AD symptoms than PBS control. Moreover, compared with pimecrolimus, cAM-MSCs effectively suppressed the degranulation of mast cells and the thickness of the dermal epidermis. Additionally, long-interval repeated subcutaneous injection of cAM-MSCs at the location of atopic dermatitis for 56 days decreased patient stress and no weight loss in comparison to pimecrolimus without causing any adverse effects. Especially, immune modulation proteins (TGF-β1, IDO1 and COX-2) were significantly increased in the cAM-MSCs treatment group. Furthermore, cAM-MSCs suppressed the proliferative capacity of effector T cells from canine PBMCs more effectively than pimecrolimus. Taken together, the results of this study proved that cAM-MSCs are effective stem cell therapy that has immunomodulation and safety for long-interval canine AD.

## Electronic supplementary material

Below is the link to the electronic supplementary material.

Supplementary Material 1

## Data Availability

Data is provided within the manuscript or supplementary information files.
