# Supplementary material for: Therapeutic effect of long-interval repeated subcutaneous administration of canine amniotic membrane-derived mesenchymal stem cells in atopic dermatitis mouse model
Source: BMC Vet Res. 2025 Feb 27;21:115. doi: 10.1186/s12917-025-04554-w (PMC11866708; doi:10.1186/s12917-025-04554-w)
Supplement: Supplementary file 1 — Supplementary Material 1 [file 12917_2025_4554_MOESM1_ESM.pdf]

Supplementary Figures

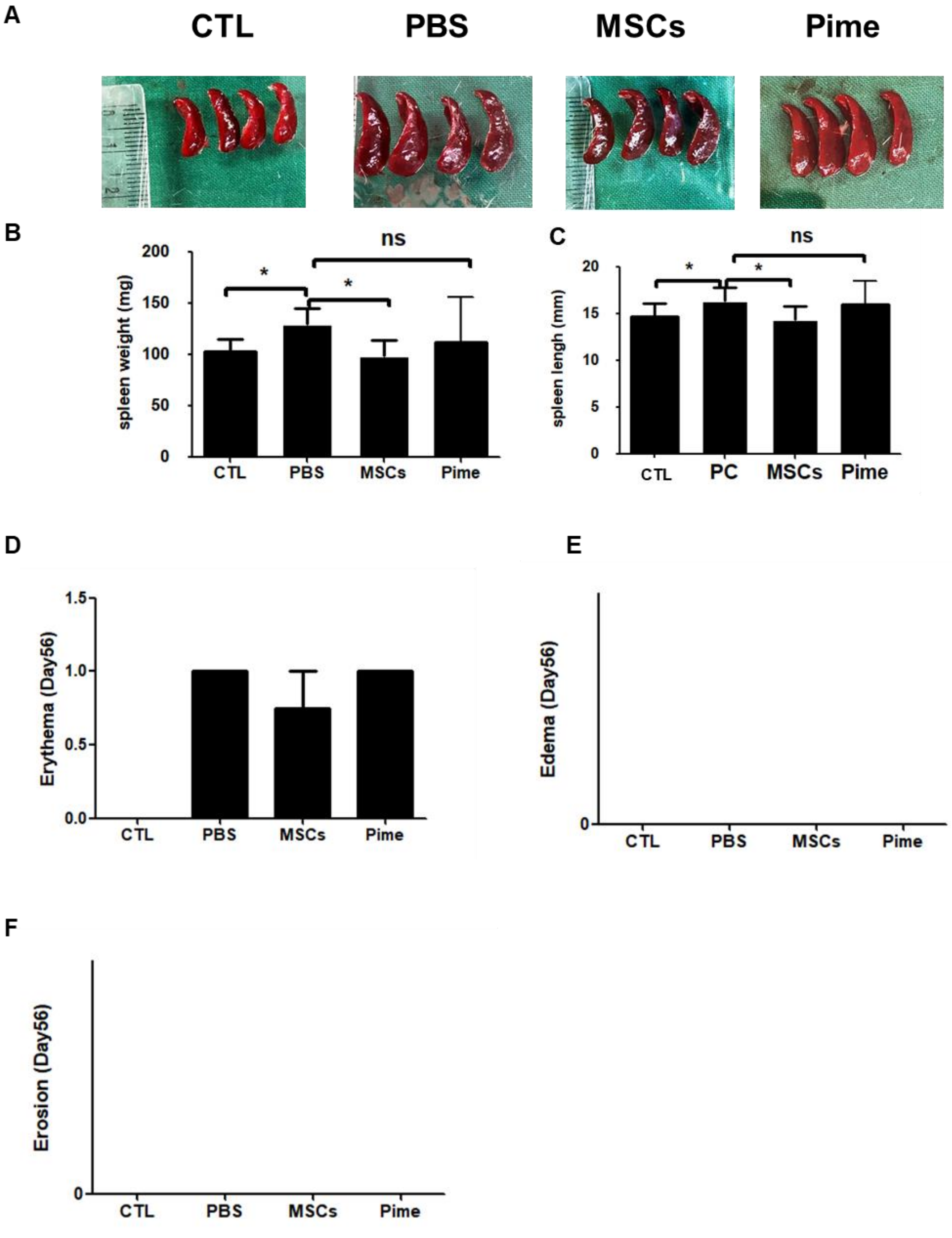

Supplementary Figure 1. (A-C) Representative spleen images (A) of CTLs, PBS, MSCs, and Pime, Quantifications of spleen weight (B) and length (C). n = 4 in each spleen; mean±SD; * $p < 0.05$, n.s. not significantly different versus the control. (D-F) the severity scores of erythema (D), edema (E), and erosion (F) for CTL, PBS, MSCs and Pime on Day 56 (none, 0; mild, 1; moderate, 2; and severe, 3).

Supplementary Figures

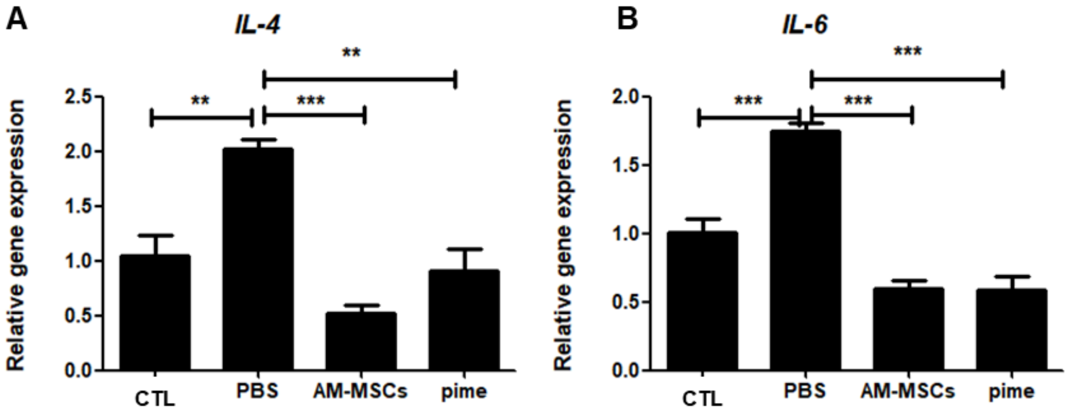

Supplementary Figure 2.(A, B) Gene expression related to inflammation: *IL*-4 (A) and *IL-6* (B). Three independent analyses were conducted for each gene.

Supplementary Tables

Supplementary Table 1.List of primers for Real time PCR.

Gene	Target	Sequence(5'→3', forward)	Sequence(5'→3', reverse)	Temp(℃)
**IL-6**	Mouse	AGTGAGGAACAAGCCAGAGC	AGCTGCGCAGAATGAGATGA	60℃
**IL-4**	Mouse	TGCTAGGCAAGCAGGAGGTGAT	GTGGCACCATAGGCATAATCTGG	60℃
**GAPDH**	Mouse	GGCATTGCTCTCAATGACAA	TGTGAGGGAGATGCTCAGTG	60℃

Supplementary Table 2.List of antibodies.

Antibody	Working Dilutions	supplier	Catalog Number	Method
**COX-2**	1 : 500	Abcam	Cat. #ab15191	IHC
**IDO-1**	1 : 500	Merck	Cat. # 05-840	IHC
**TGF-β1**	1 : 500	Abcam	Cat. # ab27969	IHC
**Mast cell tryptase**	1: 500	Abcam	Cat. # ab2378	IHC
**CD31/PECAM-1**	1: 500	R&D systems	Cat. # AF3628	IHC
**CD90**	1 : 500	Invitrogen	Cat.#12-5900-42	IHC
**Dapi**	1 : 1000	Merck	Cat. #D9542	IHC
